# The Prognostic and Mutational Characteristics of Multiple Early-stage Lung Cancers Manifesting as Subsolid Nodules

**DOI:** 10.2174/0115734056450668260615115209

**Published:** 2026-06-18

**Authors:** Bin Wang, Jiabi Zhao, Xiwen Sun, Yangyang Sun

**Affiliations:** 1 Department of Radiology, Shuguang Hospital Affiliated to Shanghai University of Traditional Chinese Medicine, Shanghai, China; 2 Department of Radiology, Zhongshan Hospital, Fudan University, Shanghai, China; 3 Department of Radiology, Shanghai Pulmonary Hospital, Tongji University School of Medicine, Shanghai, China; 4 Department of Radiology, Shanghai East Hospital, Tongji University School of Medicine, Shanghai, China

**Keywords:** Computed tomography, Lung neoplasms, Overall survival, Recurrence-free survival, Epidermal growth factor receptor, Tyrosine kinase inhibitors

## Abstract

**Introduction/Objective:**

To explore the prognostic and EGFR mutational characteristics of patients with multiple lung subsolid nodules (SSNs) after surgical resection.

**Methods:**

A retrospective analysis was performed on 250 patients with pathologically confirmed T1aN0M0 lung cancer, who were divided into single SSN (n = 126), multiple SSNs (n = 42), and single solid nodule (n = 82) groups. All patients underwent a single surgical resection and were followed up to assess recurrence-free survival (RFS) and overall survival (OS). EGFR mutation testing was performed in 131 patients.

**Results:**

The 5-year OS rates were 100% (single SSN group), 95.2% (multiple SSNs group), and 89.0% (solid nodule group) (single *vs.* multiple SSNs, *P* < 0.05; multiple SSNs *vs.* solid nodules, *P* = 0.226). The 5-year RFS rates were 99.2%, 88.1%, and 76.8%, respectively (single *vs.* multiple SSNs, *P* < 0.01; multiple SSNs *vs.* solid nodules, *P* = 0.111). EGFR mutation rates were 69.2%, 71.4%, and 42.1% in the three groups, respectively.

**Discussion:**

Patients with multiple subsolid nodules on CT had a slightly worse prognosis than those with a single subsolid nodule. It may be necessary to reconsider the rationale for simply classifying all multiple subsolid nodules as multiple primary lung cancers (MPLC). The high EGFR mutation rate in the multiple subsolid nodule group suggests that targeted therapy may be a potential treatment option for patients with multiple subsolid lesions.

**Conclusion:**

CT-detected multiple subsolid nodules are associated with a slightly poorer prognosis than single subsolid nodules, resist simplistic classification as multiple primary lung cancers, and exhibit a high EGFR mutation rate that supports targeted therapy as a treatment option.

## 1 INTRODUCTION

Lung tumors are clinically categorized into non-small cell lung cancer (NSCLC) and small cell lung cancer (SCLC). As the predominant type of lung malignancies, NSCLC is most frequently composed of adenocarcinoma [1]. The number of patients diagnosed with multiple primary lung cancers (MPLC) has been on the rise with the widespread use of thoracic computed tomography (CT) screening [2-4]. However, the diagnosis and treatment of MPLC remain highly controversial. Despite the availability of various imaging, pathological, genomic, and molecular examinations, distinguishing MPLC from intrapulmonary metastasis remains challenging due to the significant heterogeneity of lung cancer [5, 6].

In clinical practice, a large proportion of newly detected early-stage lung cancers present as subsolid nodules on computed tomography (CT). Notably, the number of patients with multiple lung subsolid nodules is also growing, yet a consensus on their diagnosis has not been reached. The 8th edition of the Tumor-Node-Metastasis (TNM) staging system for lung cancer classifies patients with multiple lung subsolid nodules as having multiple lung cancer nodules with prominent ground glass or lepidic (GG/L) features [7]. Patients in this category typically exhibit excellent outcomes with low recurrence and metastasis rates, hence are considered to have MPLC rather than intrapulmonary metastasis [8]. The 8th TNM staging system also recommends that multiple GG/L tumors be staged based on the highest T category of the nodules, with a combined N and M category [7]. Additionally, the 2017 Fleischner Guidelines suggest that the management of patients with multiple subsolid nodules should be guided by the most suspicious nodule [9]. While these guidelines and recommendations generally classify multiple subsolid nodules as MPLC, there is a lack of sufficient clinical data to further validate this conclusion.

The treatment of multiple lung subsolid nodules is also a persistent clinical dilemma. Surgical resection of all lesions is often impractical, and some patients are unwilling to undergo multiple surgical procedures. EGFR mutations are the most common genetic alterations in lung adenocarcinoma, and the efficacy of EGFR tyrosine kinase inhibitors (TKIs) in treating subsolid lesions has been investigated [10-12]. These studies have demonstrated the effectiveness of this targeted therapy for lung subsolid lesions, but determining the EGFR mutation status is crucial.

In this study, we aimed to investigate the prognostic and mutational characteristics of early-stage (clinical T1aN0M0) lung cancer, including patients with single or multiple subsolid nodules and those with solitary solid nodules. Multiple solid lung cancers, which are more likely to represent intrapulmonary metastasis [7, 13] were excluded from the study.

## 2 MATERIALS AND METHODS

### 2.1 Patients

This study was approved by the Ethics Committee of Shanghai Pulmonary Hospital (approval No. K20-025Y). Owing to its retrospective design, written informed consent from patients was waived. The study strictly adhered to the ethical standards outlined in the Declaration of Helsinki.

Electronic medical records were searched for patients who underwent surgery for diagnosed or suspected lung cancer between January 2017 and January 2018. The inclusion and exclusion criteria were as follows. Inclusion criteria: (1) The availability of chest CT images within one month prior to surgery; (2) For subsolid nodules, the solid component was ≤ 1.0 cm (clinical T1a stage), regardless of the total nodule size. For patients with multiple subsolid nodules, all nodules met this criterion; (3) Solitary solid nodules with a maximum diameter ≤ 1.0 cm; (4) Maximum diameter of subsolid and solid nodules > 5 mm (nodules ≤ 5 mm are almost always benign); (5) CT images with a slice thickness < 2 mm; (6) The minimum follow-up duration of 60 months. Exclusion criteria: (1) Preoperative evidence of lymph node involvement or distant metastasis based on comprehensive evaluation; (2) Multiple solid nodules or a combination of subsolid and solid nodules. The measurement of the solid component and total nodule size was performed in accordance with the recommendations of the International Association for the Study of Lung Cancer (IASLC) and the Fleischner Society [14, 15].

### 2.2 Classification of Patients by CT Images

All enrolled patients were divided into three groups based on preoperative chest CT findings. (1) Single subsolid group: Patients with only one subsolid nodule; (2) Multiple subsolid group: Patients with two or more subsolid nodules; (3) Single solid group: Patients with only one pure solid nodule. Two radiologists conducted the classification. In cases of disagreement, a senior radiologist was consulted to reach a consensus.

### 2.3 Surgical Resection and Pathological Diagnosis

The surgical approach was determined based on tumor size, location, CT features, and intraoperative frozen section diagnosis. Furthermore, patients' pulmonary function and general health status were also considered in selecting the surgical procedure. Lobectomy with systematic lymph node sampling was the primary surgical procedure, while sublobar resection (including wedge resection and segmentectomy) with or without lymph node resection was performed for the remaining patients. All included patients underwent only one surgical resection during the follow-up period, including those with multiple subsolid nodules. Pathological evaluation and histological subtype classification of resected specimens were mainly performed in accordance with the International Association for the Study of Lung Cancer/American Thoracic Society/European Respiratory Society (IASLC/ATS/ERS) classification of lung adenocarcinoma [16].

### 2.4 Follow-up and Prognosis Evaluation

Follow-up intervals were 3–6 months for the first 2 years, followed by annual examinations thereafter. Follow-up assessments mainly included physical examination, chest CT, and relevant tumor marker tests. Brain magnetic resonance imaging (MRI), radionuclide bone scan, or positron emission tomography-computed tomography (PET-CT) were selectively performed for patients with specific symptoms or suspected recurrence. All patients were followed up for at least 5 years after the last surgery.

### 2.5 Examination of EGFR Mutation Status

DNA was extracted from formalin-fixed paraffin-embedded (FFPE) tumor tissue. The EGFR pharmDX kit was used to detect EGFR protein expression, and results were scored and interpreted according to standard human EGFR 2 scoring guidelines. EGFR mutation status was analyzed at exons 18, 19, 20, and 21 using a polymerase chain reaction (PCR)-based assay as recommended in previous literature [17], and results were further confirmed by DNA sequencing.

### 2.6 Statistical Analysis

Quantitative data were presented as mean ± standard deviation (SD) or median [25^th^–75^th^ percentile]; qualitative data were presented as n (%). The chi-square test or Fisher's exact test was used for comparisons of qualitative variables between groups, and the t-test or Wilcoxon test was used for comparison of quantitative data. Recurrence-free survival (RFS) and overall survival (OS) were calculated, and differences in RFS or OS between groups were evaluated using the Kaplan–Meier method. All statistical analyses were performed using SPSS statistical software (version 22.0, IBM, Armonk, NY, USA). A two-sided P-value < 0.05 was considered statistically significant.

## 3 RESULTS

### 3.1 Characteristics of the Study Group

A total of 250 patients were finally enrolled in the study, including 168 patients with subsolid nodules and 82 patients with single solid nodules. The patient selection and inclusion process are shown in Fig. (**1**), and some representative cases are shown in Fig. (**2**). Baseline characteristics of enrolled patients are presented in Tables **1** and **2**. Previous studies have confirmed that the diameter of the solid component, rather than the total nodule size, is more closely correlated with the prognosis of patients with subsolid nodules [18-21]; therefore, the diameter of the solid component was measured to represent the size of subsolid nodules. As shown in Table **1**, the subsolid nodule group was more likely to be diagnosed with low-risk pathological subtypes, undergo lobectomy, and have a higher EGFR mutation rate compared with the solid nodule group.

### 3.2 Comparison between Patients with Single or Multiple Subsolid Nodules

Patients with subsolid nodules were further divided into the single subsolid nodule group (n = 126) and the multiple subsolid nodule group (n = 42). As shown in Table **2**, patients with multiple subsolid nodules had a significantly higher rate of lymph node metastasis compared with those with single subsolid nodules (9.6% *vs.* 0.0%, *P* < 0.01).

Other baseline characteristics, such as total nodule size, solid component size, and surgical procedure, showed no significant differences between the two groups, indicating good consistency in baseline data and minimal influence of confounding variables.

### 3.3 Prognosis Analysis

The median follow-up duration was 54 months. Among all enrolled patients, 25 (9%) experienced recurrence, and 11 (4%) died, respectively. No deaths were observed in the single subsolid nodule group during follow-up. Of the 11 deaths, 2 occurred in the multiple subsolid nodule group and 9 in the single solid nodule group. Recurrence was observed in 1 patient in the single subsolid nodule group, 5 in the multiple subsolid nodule group, and 19 in the single solid nodule group.

**Fig. (3):**
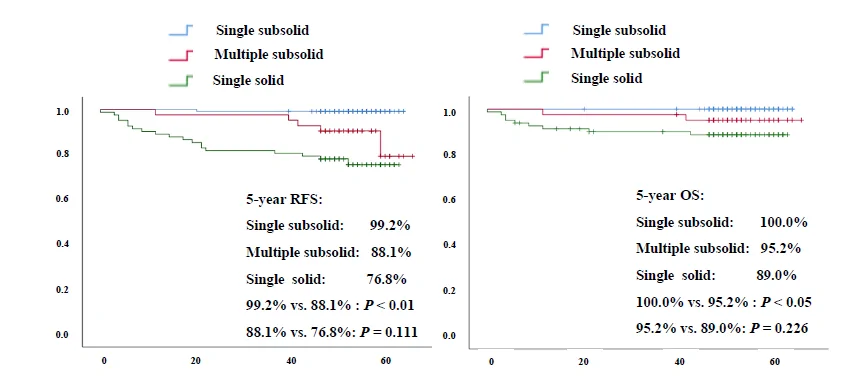
Kaplan-Meier curves based on the groups of single subsolid, multiple subsolid and single solid nodules. RFS, recurrence-free survival; OS, overall survival.

The 5-year OS rates were 100%, 95.2%, and 89.0% in the single subsolid nodule group, multiple subsolid nodule group, and single solid nodule group, respectively (100% *vs.* 95.2%, *P* < 0.05; 95.2% *vs.* 89.0%, *P* = 0.226). The 5-year RFS rates were 99.2%, 88.1%, and 76.8% in the single subsolid nodule group, multiple subsolid nodule group, and single solid nodule group, respectively (99.2% *vs.* 88.1%, *P* < 0.01; 88.1% *vs.* 76.8%, *P* = 0.111) (Fig. **3**).

**Fig. (4):**
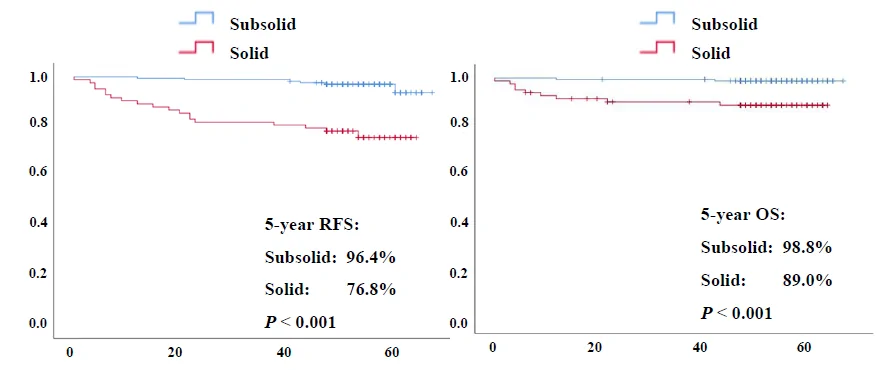
Kaplan-Meier curves based on the groups of subsolid and solid nodules. **Note:** RFS, recurrence-free survival; OS, overall survival.

Furthermore, we compared the survival outcomes between the subsolid nodule group (including single and multiple subsolid nodules) and the single solid nodule group. Both OS and RFS showed significant differences between the two groups. Patients in the subsolid nodule group had better 5-year OS (98.8% *vs.* 89.0%, *P* < 0.001) and 5-year RFS (96.4% *vs.* 76.8%, *P* < 0.001) (Fig. **4**), even though all patients in both groups were classified as clinical T1aN0M0.

### 3.4 EGFR Mutation Status

**Fig. (5):**
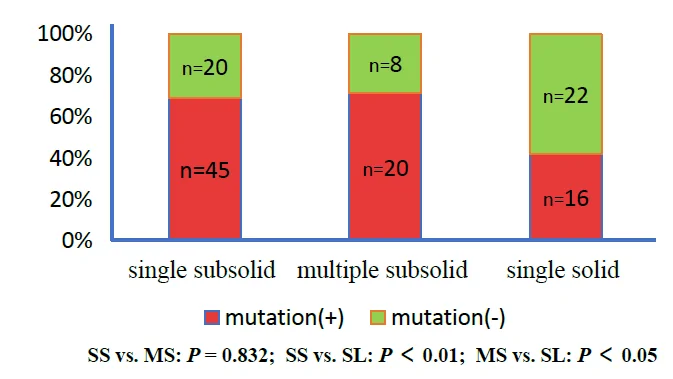
Epidermal growth factor receptor (EGFR) mutations in the groups of single subsolid, multiple subsolid and single solid nodules. **Note:** SS, single subsolid; MS, multiple subsolid; SL, single solid.

Among 131 patients with stage I lung adenocarcinoma who underwent EGFR mutation testing, EGFR mutations were identified in 81 cases (61.8%), with mutation rates of 69.2% (45/65) in the single subsolid nodule group, 71.4% (20/28) in the multiple subsolid nodule group, and 42.1% (16/38) in the single solid nodule group. EGFR mutation positivity was comparable between single subsolid and multiple subsolid nodules (*P* = 0.832), while both subgroups exhibited a significantly higher mutation rate than single solid nodules (*P*<0.01 and *P*<0.05, respectively; Fig. **5**). This indicates that subsolid nodule morphology—irrespective of multiplicity—is associated with a higher prevalence of EGFR mutations, providing a morphological reference for molecular stratification in early-stage lung adenocarcinoma. We performed a comparative prognostic analysis of EGFR mutation-positive and -negative patients, with results presented in Table **3** and Fig. (**6**). Table **3** shows no significant differences in recurrence or mortality between the two groups. Similarly, our Kaplan-Meier analysis of RFS revealed 5-year RFS rates of 94.0% (mutation-negative) and 88.9% (mutation-positive), with no statistically significant difference (*P*=0.337). Consistent with previous systematic analyses [22], EGFR mutation status has been widely reported to be associated with survival outcomes in lung adenocarcinoma, though our study did not reach a statistically significant difference, which may be related to the limited sample size and follow-up period.

## 4 DISCUSSION

Lung cancer remains the most common malignant tumor and the leading cause of cancer-related death worldwide [23]. Published studies have reported that multiple primary lung cancers account for approximately 3.7% to 8.4% of surgically resected non-small cell lung cancers (NSCLC) [24, 25], and this proportion may be much higher in subsolid lung cancers. In this study, 42 of 168 (25%) patients with subsolid nodules were diagnosed with multiple subsolid lung cancers. Distinguishing MPLC from intrapulmonary metastasis in these patients remains challenging due to the significant histological heterogeneity of lung cancer [5]. However, due to their excellent outcomes and low recurrence and metastasis rates, multiple subsolid nodules are generally considered to be MPLC rather than intrapulmonary metastasis [8].

In this study, the 5-year OS rates were 100% and 95.2% in the single subsolid nodule group and multiple subsolid nodule group, respectively (*P* < 0.05), and the 5-year RFS rates were 99.2% and 88.1%, respectively (*P* < 0.01). Despite being classified in the same TNM clinical stage, patients with single subsolid nodules had better OS and RFS than those with multiple subsolid nodules. This finding provides new insights into multiple lung subsolid nodules and underscores the need for careful clinical management. Additionally, the rationale for simply classifying all patients with multiple subsolid nodules as MPLC should be reconsidered, given the prognostic differences observed.

Numerous studies have demonstrated that the size of the solid component is a more reliable prognostic predictor for patients with subsolid nodules. The new clinical T stage classification is based on the size of the solid component, excluding the ground-glass opacity (GGO) component [15]. According to this new staging system, subsolid nodules with a solid component ≤ 1 cm and pure solid nodules with a total size ≤ 1 cm are both classified as cT1a, implying similar prognoses. However, in our previous study [26], we demonstrated that patients with subsolid nodules had better 5-year OS and RFS than those with solid nodules, despite both groups being classified as cT1aN0M0.

In the current study, we further compared the survival outcomes between the subsolid nodule group (including single and multiple subsolid nodules) and the solid nodule group, with all patients classified as clinical T1aN0M0. The results showed that patients in the subsolid nodule group had significantly better 5-year OS (98.8% *vs.* 89.0%, *P* < 0.001) and 5-year RFS (96.4% *vs.* 76.8%, *P* < 0.001) than those in the single solid nodule group, which is consistent with our previous findings.

Although multiple subsolid nodules are mostly recognized as multiple primary lung cancer (MPLC) with a favorable prognosis, their clinical management still poses considerable challenges. Patients with multiple subsolid nodules are often unable to tolerate radical resection of all lesions owing to restricted pulmonary reserve and surgical trauma. Lung adenocarcinoma with EGFR mutations responds positively to EGFR-TKIs, and systematic analyses have verified that EGFR-TKI therapy has achieved substantial progress in the treatment of lung tumors [27]. Several studies [10-12] have shown that subsolid nodules also respond to EGFR-TKI treatment. In this study, resected lesions from patients with multiple subsolid nodules exhibited a high EGFR mutation rate, suggesting that targeted therapy may be a viable option for these patients.

## 5 LIMITATIONS

This study has several limitations. First, the data were derived from a single institution, and the retrospective design made it difficult to control for confounding prognostic factors. A validation cohort study will be required to further confirm our conclusions. Second, the sample size, particularly the number of patients with multiple subsolid nodules, was relatively small, which may have affected the accuracy and stability of the results. Additionally, the detailed causes and mechanisms underlying the prognostic differences between patients with single and multiple subsolid nodules were not fully investigated or explained. We aim to address this interesting and challenging question in future research.

## CONCLUSION

This study found that despite being in the same clinical stage, patients with multiple subsolid nodules had a slightly worse prognosis than those with single subsolid nodules, and multiple subsolid nodules exhibited a high EGFR mutation rate. These findings may provide valuable information for the management and treatment of the growing number of patients with multiple lung subsolid nodules.

## Figures and Tables

**Fig. (1):**
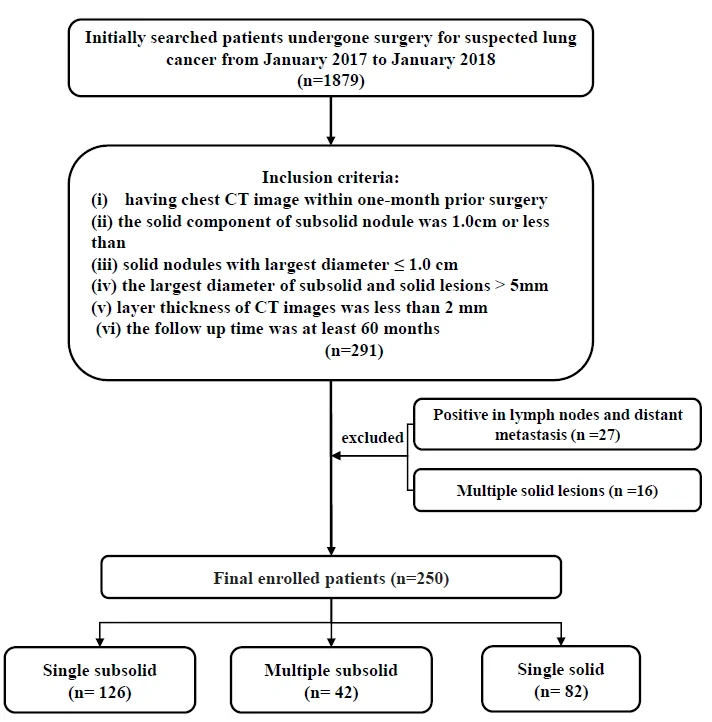
Patients’ selection.

**Fig. (2):**
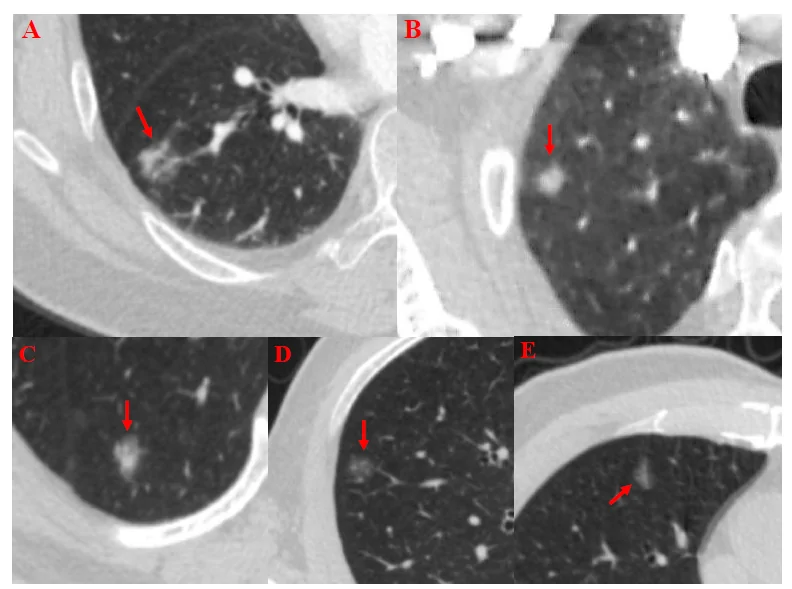
Examples of the enrolled patients. (**A**) patient with one single subsolid nodule. (**B**) patient with one single pure solid nodule. (**C**-**E**) were from one patient, one part-solid and two pure ground-glass nodules.

**Fig. (6):**
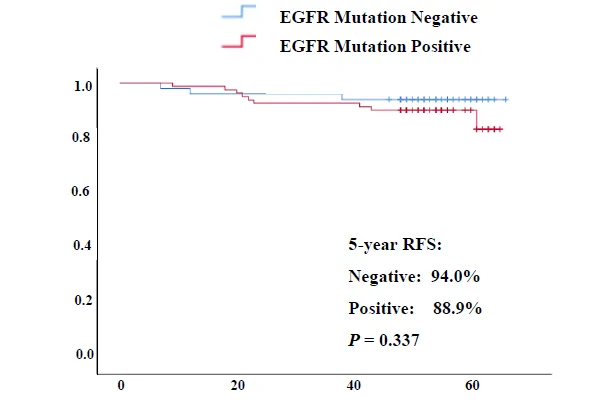
Recurrence-free survival in EGFR mutation-positive versus mutation-negative patients. RFS, recurrence-free survival; EGFR, epidermal growth factor receptor.

**Table 1:** Basic information of all enrolled patients divided into subsolid and solid groups.

**Variables**	**Subsolid(n=168)**	**Solid(n=82)**	***P***
**Age**	59.2±9.1	57.7±11.1	**0.279**
**Sex (Female)**	103(61.3%)	41(50.0%)	**0.089**
**Solid component/ total diameter (mm)**	0.6[0.6-0.7]	0.9[0.8-1.0]	**/**
**Smoking history (Yes)**	25(14.9%)	16(19.5%)	**0.353**
**Operative procedure**			**<0.05**
Lobectomy	144(85.7%)	61(74.4%)	
Sublobar resection	24(14.3%)	21(25.6%)	
**Pathology type**			**<0.001**
Low risk	89(53.0%)	7(8.5%)	
Medium risk	76(45.2%)	55(67.1%)	
High risk	1(0.6%)	9(11.0%)	
Non-adenocarcinoma	2(1.2%)	11(13.4%)	
**EGFR Mutation**			**<0.01**
Positive	65(69.9%)	16(42.1%)	
Negative	28(30.1%)	22(57.9%)	
Not tested	75(/)	44(/)	
**Pathological nodal involvement**			**0.057**
N0	164(97.6%)	75(91.5%)	
N1/N2	4(2.4%)	7(8.5%)	

**Note:** Qualitative variables are described as n (%); quantitative variables are described as mean ±SD or median (25^th^-75^th^).

Low risk: Lepidic-predominant or preinvasive adenocarcinoma; Medium risk: Papillary/acinar-predominant adenocarcinoma; high risk: Contains micropapillary or solid component adenocarcinoma (≥ 5%). EGFR: epidermal growth factor receptor.

**Table 2:** Basic information of enrolled subsolid patients divided into single subsolid and multiple subsolid groups.

**Variables**	**Single Subsolid** **(n=126)**	**Multiple Subsolid (n=42)**	***P***
**Age**	58.7±8.8	60.9±9.8	**0.178**
**Sex (Female)**	72(57.1%)	31(73.8%)	**0.055**
**Smoking history (Yes)**	19(15.1%)	6(14.3%)	**0.900**
**Total size**	1.9[1.5-2.5]	1.95[1.7-2.6]	**0.547**
**Solid component (mm)**	0.6[0.6-0.7]	0.6[0.6-0.8]	**0.388**
**Operative procedure**			**0.309**
Lobectomy	110(87.3%)	34(81.0%)	
Sublobar resection	16(12.7%)	8(19.0%)	
**Pathology type**			**0.090**
Low risk	62(49.2%)	27(64.3%)	
Medium/high risk	64(50.8%)	15(35.7%)	
**EGFR Mutation**			0.832
Positive	45(69.2%)	20(71.4%)	
Negative	20(30.8%)	8(28.6%)	
Not tested	61(/)	14(/)	
**Pathological nodal involvement**			**0.003**
N0	126(100.0%)	38(90.5%)	
N1/N2	0(0.0%)	4(9.6%)	

**Note:** Qualitative variables are described as n (%); quantitative variables are described as mean ±SD or median (25^th^-75^th^).

Low risk: lepidic predominant invasive adenocarcinoma or preinvasive adenocarcinoma; Medium risk: acinar and papillary predominantly invasive adenocarcinoma; High risk: invasive adenocarcinoma containing micropapillary or solid components (≥5%). EGFR: epidermal growth factor receptor.

**Table 3:** Correlation between prognosis and EGFR mutation.

**Prognosis**	**EGFR Mutation**		***P***
	**Positive (n=81)**	**Negative (n=50)**	
**Recurrence**			**0.372**
Yes	9(11.1%)	3(6.0%)	
**No**	72(88.9%)	47(94.0%)	
**Death**			**0.862**
Yes	2(2.5%)	1(2.0%)	
No	79(97.5%)	49(98.0%)	

**Note:** EGFR: epidermal growth factor receptor.

## Data Availability

The data for this study can be obtained from the corresponding authors [X.S] and [Y.S] using the contact information provided in the manuscript.

## LIST OF ABBREVIATIONS

CT: Computed Tomography
OS: Overall Survival
RFS: Recurrence-Free Survival
MPLC: Multiple Primary Lung Cancer
c-T1aN0M0: clinical T1aN0M0
IASLC: The International Association for the Study of Lung Cancer
SD: Standard Deviation
TNM: Tumor, Node, and Metastasis
GG/L: Ground Glass or Lepidic
EGFR: Epidermal Growth Factor Receptor
TKIs: Tyrosine Kinase Inhibitors
MRI: Magnetic Resonance Imaging

## STANDARDS OF REPORTING

STROBE guidelines were followed.
